# Small, but surprisingly repetitive genomes: transposon expansion and not polyploidy has driven a doubling in genome size in a metazoan species complex

**DOI:** 10.1186/s12864-019-5859-y

**Published:** 2019-06-07

**Authors:** J. Blommaert, S. Riss, B. Hecox-Lea, D. B. Mark Welch, C. P. Stelzer

**Affiliations:** 1 Research Department for Limnology, University of Innsbruck, Mondsee, Austria; 2 Josephine Bay Paul Center for Comparative Molecular Biology and Evolution, Marine Biological Laboratory, Woods Hole, MA USA

**Keywords:** Genome expansion, Cryptic species complex, Polyploidy, Transposable elements, C-value

## Abstract

**Background:**

The causes and consequences of genome size variation across Eukaryotes, which spans five orders of magnitude, have been hotly debated since before the advent of genome sequencing. Previous studies have mostly examined variation among larger taxonomic units (e.g., orders, or genera), while comparisons among closely related species are rare. Rotifers of the *Brachionus plicatilis* species complex exhibit a seven-fold variation in genome size and thus represent a unique opportunity to study such changes on a relatively short evolutionary timescale. Here, we sequenced and analysed the genomes of four species of this complex with nuclear DNA contents spanning 110–422 Mbp. To establish the likely mechanisms of genome size change, we analysed both sequencing read libraries and assemblies for signatures of polyploidy and repetitive element content. We also compared these genomes to that of *B. calyciflorus*, the closest relative with a sequenced genome (293 Mbp nuclear DNA content).

**Results:**

Despite the very large differences in genome size, we saw no evidence of ploidy level changes across the *B. plicatilis* complex. However, repetitive element content explained a large portion of genome size variation (at least 54%). The species with the largest genome, *B. asplanchnoidis,* has a strikingly high 44% repetitive element content, while the smaller *B. plicatilis* genomes contain between 14 and 25% repetitive elements. According to our analyses, the *B. calyciflorus* genome contains 39% repetitive elements, which is substantially higher than previously reported (21%), and suggests that high repetitive element load could be widespread in monogonont rotifers.

**Conclusions:**

Even though the genome sizes of these species are at the low end of the metazoan spectrum, their genomes contain substantial amounts of repetitive elements. Polyploidy does not appear to play a role in genome size variations in these species, and these variations can be mostly explained by changes in repetitive element content. This contradicts the naïve expectation that small genomes are streamlined, or less complex, and that large variations in nuclear DNA content between closely related species are due to polyploidy.

**Electronic supplementary material:**

The online version of this article (10.1186/s12864-019-5859-y) contains supplementary material, which is available to authorized users.

## Background

Genome size varies greatly across eukaryotic organisms, spanning five orders of magnitude [1]. Here, following Greilhuber [2], we use the term genome size to refer to the holoploid genome size, the total amount of DNA in a eukaryotic nucleus, rather than the DNA content of a gamete nucleus (the C-value), which is often used as a synonym for genome size. It has become widely acknowledged that, in eukaryotes, genome size does not correlate with so-called “organismal complexity”, or even with gene number. These puzzling observations have been summarized under the term “C-value Enigma” [3] and still comprise a major problem in evolutionary biology.

Many efforts to understand the causes of changes in DNA content have focused on ploidy level variation and broad interspecific genome size changes, especially in regards to species divergence and adaptive radiations in plants [4]. For example, in the genus *Tabebuia* and its sister groups, genome size varies approximately 4-fold, with much of this variation explained by polyploidy and other chromosome level changes [5]. Genome size variations on shorter evolutionary timescales (such as between closely related species) are also well known in plants [4, 6], and often involve changes in ploidy level and sometimes varying amounts of non-coding DNA [7–9]. For example, in a genus of carnivorous plants, genome size varies up to 25-fold, with polyploidy responsible for the larger changes in genome size, and repetitive element loss and gain responsible for smaller scales of genome size change [10]. Yang et al. found that intron loss played a role in genome size reduction between two *Arabidopsis* species [11]. Studies of genome size variation in animals tend to focus on more distantly related taxa [1]. For example a recent study on genome size evolution in birds and mammals found that DNA gain from transposons was counteracted in many cases by DNA loss by segmental deletions [12]. Another recent study examined the evolution of polyploidy and transposable element dynamics across catfish. The authors identified two polyploidy events in the history of this family, and found that transposable element content was influenced by these ploidy changes across the species studied [13]. Examples of genome size variation on closer evolutionary scales have been identified in a few animals species [14–16], but detailed genomic examination of these cases is rare. Some species of snapping shrimp have been found to exhibit genome size variation that does not appear to be caused by polyploidy, but has not been further characterised [17]. Other examples include genome size change in a clade of butterflies caused by an increase in transposable elements [18], and analyses of variation in the composition of B-chromosomes in grasshoppers [19–21]. These studies exemplify how the comparison of genome size and genomic composition across broad evolutionary scales can illuminate the causes of genome size variation, and highlight that polyploidy is often implicated in large genome size changes within or between closely related species, while repetitive elements tend to be linked with smaller or more gradual changes in genome size.

**Fig. 1:**
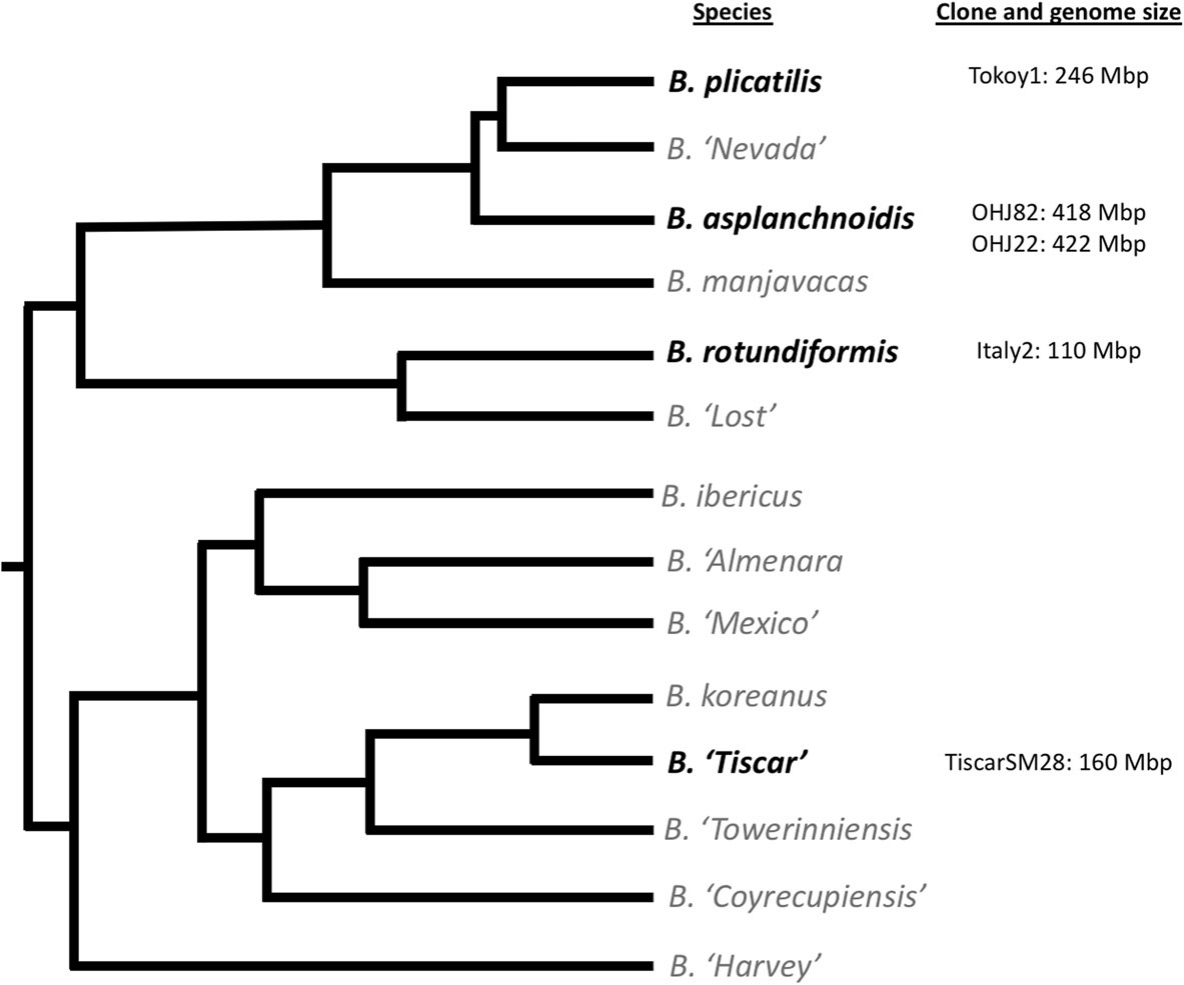
Rotifer clones used in this study and their phylogenetic relationships within the *Brachionus plicatilis* species complex. Figure redrawn and simplified from the COI and ITS1 Maximum-likelihood tree from [25]. Branch tips represent species, species included in this study are in black text, while others are in grey

Our goal is to identify the main mechanisms driving interspecific differences in genome size using comparative genomics of the *Brachionus plicatilis* species complex, a group of monogonont rotifers that exhibits large variation in genome size, both within and across species bounds despite morphological and ecological similarity [22–25]. The *B. plicatilis* complex is one of the most extensively studied rotifer groups and has long been recognized as a model of ecological adaptation and speciation [23, 25, 26]. Increasing genomic resources and tools make it a promising model for studying the evolution of genome size [27, 28]. Here we sequenced five genomes of four species from the *B. plicatilis* species complex: *B. plicatilis* sensu stricto (clone Tokyo1), *B. asplanchnoidis* (clones OHJ82 and OHJ22), *Brachionus* sp. *‘*Tiscar’(clone TiscarSM28), and *B. rotundiformis* (clone Italy2). The genome sizes of these clones were previously estimated by flow cytometry to be 246 Mbp, 418 and 422 Mbp, and 160 Mbp and 110 Mbp respectively ([22, 25], Table 1). The phylogenetic relationships among the studied clones and species are summarised in Fig. 1. After genome sequencing and assembly, we considered evidence for polyploidy and assessed repetitive element content with both read-based and genome assembly-based methods [29–31]. Additionally we compared these genome sequences to the recently published genome of *B. calyciflorus* [32], a more distantly related rotifer species with a genome size of 293 Mbp [24].

**Table 1:** Genome assembly statistics, showing the species name, clone name, genome size estimated by flow cytometry, total number of bp sequenced, assembly size, and assembly N50 (before and after contaminant removal), mean observed coverage calculated in 500 bp windows across each assembly, mean observed coverage of non-repetitive regions, expected coverage range based on mapped reads and all reads, and the % of metazoan BUSCO genes present in each assembly (either complete, duplicated, or fragmented)

			Raw Reads			After Contaminant Removal							
Species Name	Clone	Genome Size (Mbp)	Total bp Sequenced	Total Contig Length in Mbp (Incl. Scaffolds)	N50 (bp)	Total bp Sequenced	Total Contig Length in Mbp (Incl. Scaffolds)	N50 (bp)	% Unmapped Reads	Mean Observed Coverage	Mean Observed Coverage Outside Repeats	Expected Coverage Range	% Metazoan BUSCO Genes Present
*B. rotundiformis*	Italy2	110	2.06E+ 09	60	24,447	2.01E+ 09	51.06	42,810	8.78	36.21	33.63	16-18x	92%
B. sp. ‘Tiscar’	TiscarSM28	164	3.45E+ 09	84	13,443	3.39E+ 09	70.99	24,091	4.65	44.50	43.33	19-21x	92%
*B. plicatilis*	Tokyo1	246	4.72E+ 09	127	10,058	4.35E+ 09	99.5	15,643	7.87	40.06	36.18	16-18x	92%
*B. asplanchnoidis*	OHJ82	418	7.10E+ 09	116	6154	6.90E+ 09	114.8	9227	16.21	49.20	38.75	14-17x	91%
	OHJ22	422	9.77E+ 09	115	5047	9.73E+ 09	114.4	10,465	12.02	74.09	55.94	20-23x	92%

## Results

### Genome sequencing, assembly and assessment

We sequenced and assembled five genomes from four species within the *B.plicatilis* species complex: *B. rotundiformis* (Italy2), *B.* sp. *‘*Tiscar*’* (TiscarSM28), *B. plicatilis* s.s. (Tokyo1), and *B. asplanchnoidis* (OHJ82 and OHJ22). The number of sequenced base pairs (bp) ranged from 2.06 Gbp to 9.77 Gbp; we identified 0.4–8% of reads as coming from contaminants, and retained between 2.01 and 9.73 Gbp. Kmer analyses of the different cleaned read libraries revealed that the genomes of both *B. asplanchnoidis* strains (OHJ82, 0.412%; OHJ22, 0.412%) were more heterozygous than Italy2 (0.055%), TiscarSM28 (0.178%), and Tokyo1 (0.109%). The *B. calyciflorus* genome had an estimated heterozygosity of 1.66%. Assembly size for Italy2, TiscarSM28, and Tokyo1 was approximately half of the holoploid genome size, while the mean read depth across the entire assembly and in non-repetitive regions was slightly less than twice the expected coverage (Table 1). The contig N50 of these genomes, an indication of assembly contiguity, ranged from 15,643 bp in Tokyo1 to 42,810 bp in Italy2. In contrast, despite much greater sequencing effort the *B. asplanchnoidis* assemblies were about 27% of the genome size, with a mean read depth slightly more than twice the expected coverage in non-repetitive regions of the assemblies. Both the OHJ82 and OHJ22 assemblies were ~ 115 Mbp, with contig N50 values around 10,000 bp. Each of the five assemblies had 91–92% of the metazoan BUSCO genes (Table 1). Overall, 5.5% of the metazoan BUSCO genes (54 genes) were not found in any of our assemblies, and 740 genes (75.7%) were found in complete single copies in all five (Additional file 5: File S1).

### Ploidy assessment

**Fig. 2:**
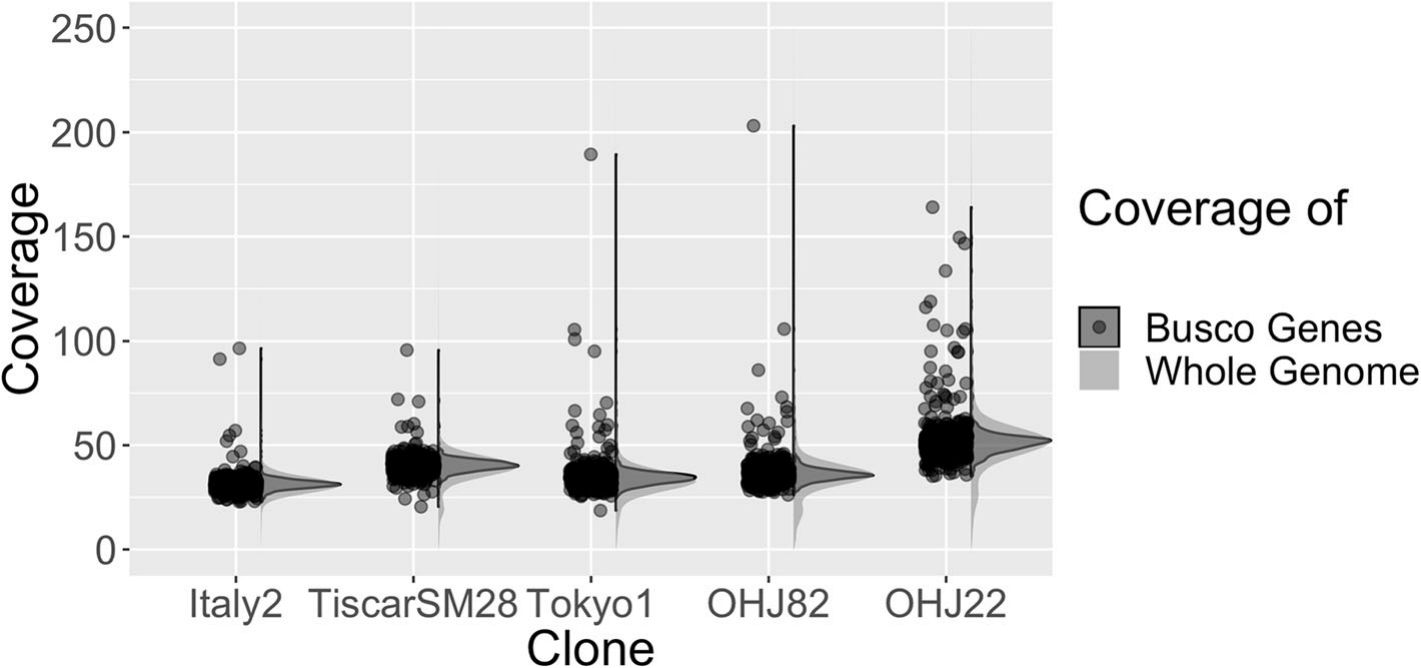
Distribution of observed coverage (on a per-gene basis) of a subset of BUSCO genes shared across all assemblies, dots indicate mean coverage values for each gene. Coverage distribution across the whole genome assemblies (in 500-bp windows) is shown in a grey overlay

Because very large changes in genome size between species often suggest changes in ploidy, we examined our assemblies for differences in read coverage and allele frequency. For all species, median observed read coverage of the non-repetitive regions of the assembly was about twice the expected coverage (Table 1). In all cases, genome coverage was unimodal, arguing against ploidy differences between species (Fig. 2). The coverage distributions of the 740 shared BUSCO genes followed the overall genome coverage in each assembly; a small fraction of genes had coverage significantly higher than the median, and there were more of these in the larger genomes (Fig. 2, Additional file 5: File S1). With the exception of Tokyo1, which had a very low number of SNPs in the BUSCO genes, the frequency distributions of minor alleles in the shared BUSCO genes were similar across species, with the frequency of most minor alleles in the 0.4–0.5 range (Additional file 1: Figure S1).

To assess coverage and allele frequency independently from assembly, we examined coverage of heterozygous kmer pairs in each read library. Comparing the relative coverage of each pair to the normalized frequency of the minor sequence can reveal patterns of ploidy and heterozygosity. For all five read libraries, the spectra indicated that most heterozygous kmers were covered around 4n, with a minor kmer relative frequency around 0.5. There was indication of a minor peak around 2n, most visible in TiscarSM28 and both *B. asplanchnoidis* libraries. The *B. calyciflorus* PE500 read library had a major peak at 2n with a minor kmer frequency of 0.5, but also an extended tail of kmer pairs with 3n and 4n coverage and minor kmer frquency of 0.3 and 0.5, respectively (Additional file 2: Figure S2). Finally, we used the program nQuire to evaluate models of diploidy, triploidy, and tetraploidy using all reads, reads that did not map to highly repetitive regions (discussed below), and reads mapping to BUSCO genes. While the “denoise” step of analysis removed at least 40% of the sites from the first two datasets, all three datasets supported a model of diploidy for Italy2, TiscarSM28, OHJ22 and OHJ82, and tetraploidy for Tokyo1 and *B. calyciflorus* (Additional file 6: File S2).

### Repetitive element analyses

**Fig. 3:**
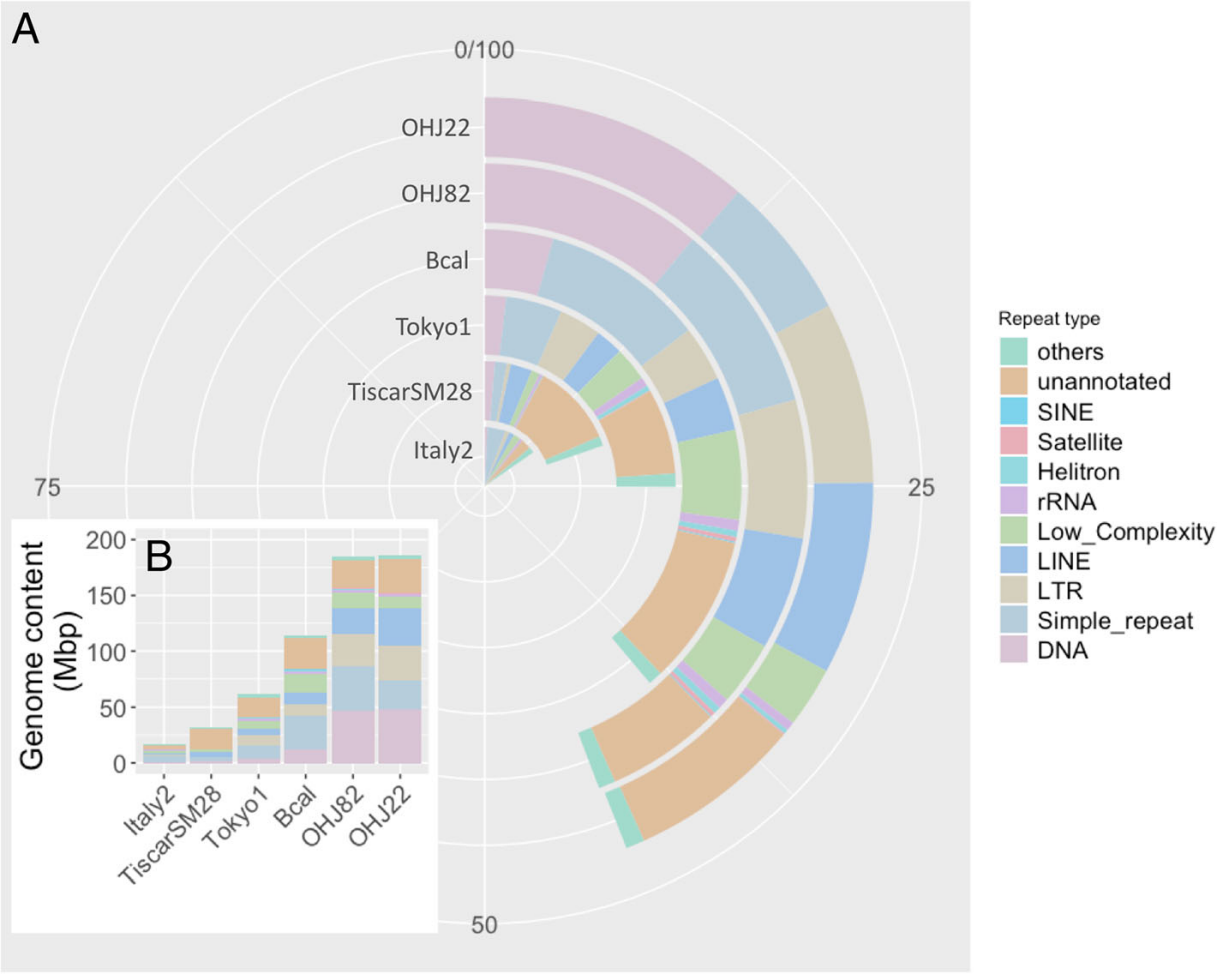
**a** Proportional repetitive element content estimates per genome using dnaPipeTE, **b** shows these estimates in Mbp of each genome, Bcal = *B. calyciflorus*

RepeatMasker, using either its “Metazoa” library or de novo RepeatModeler libraries, identified a small number of repetitive elements in each assembly (Additional file 7: File S3). Although the total repetitive DNA content increased with assembly size, the proportion of repetitive DNA only increased from 6 to 11% and did not account for significant portions of the differences in genome size across the species complex. However, de novo repetitive element identification using the program dnaPipeTE directly on read libraries revealed more repetitive elements, in terms of both diversity and genome proportion (Fig. 3, Additional file 7: File S3). Estimates of the genome content of these elements consistently and significantly increased with genome size in both absolute (linear regression, *p* = 0.0014, df = 4) and relative amounts (linear, regression, *p* = 0.0003, df = 4), from 16.8 Mbp in Italy2 (15%) to 185.92 Mbp in OHJ22 (44%). The difference in repetitive content between Italy2 and OHJ22 was just over half (54%) of the total difference in genome size (Fig. 3). Repetitive elements could account for 71% of the genome size difference between OHJ82 and Tokyo1 (the most closely related species to *B. asplanchnoidis*). When the repetitive elements generated from this method were used as a library for RepeatMasker, similar, but slightly lower proportions of the genome assemblies were annotated as repetitive (Additional file 7: File S3).

**Fig. 4:**
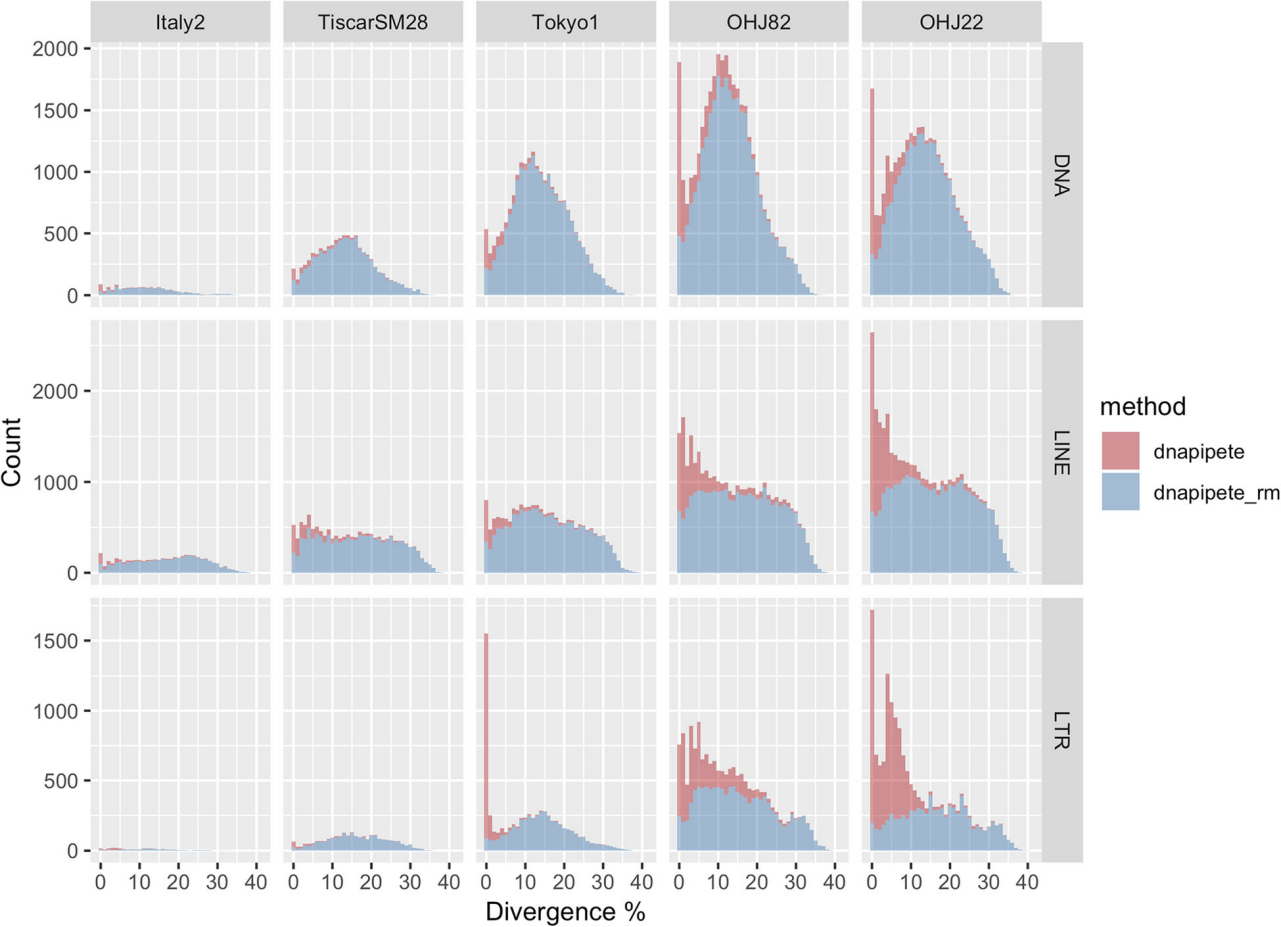
Distributions of repetitive element divergence estimates of three repetitive element classes from repetitive element annotation of read libraries (dnaPipeTE, red) and assemblies (dnaPipeTE_RM, blue). For dnaPipeTE the count reflects the number of reads which had a BLAST hit to any one dnaPipeTE assembled repetitive element, and for dnaPipeTE_RM, this represents one instance of a BLAST alignment of a dnaPipeTE assembled repetitive element in the respective genome assembly

LTR (Long Terminal Repeat) and LINE (Long Interspersed Nuclear Element) retrotransposons, and DNA transposons are the three largest groups of annotated transposons in the *B. asplanchnoidis* genomes. Together, these account for 3.3% of the genome of Italy2 and 27% of the genome in OHJ22 (Fig. 3). Additionally, as genome size increases across the species complex, the number of less diverged elements in these three groups increases, and this increase is not observed when considering only assembly-based repeat annotation (Fig. 4). The proportion of less diverged elements in these classes also increases with genome size (Additional file 3: Figure S3). Within *B*. *asplanchnoidis* (OHJ82 and OHJ22), there are also changes in the number and proportion of less diverged elements.

Using the dnaPipeTE method we estimated that the *B. calyciflorus* genome consists of 38.9% repetitive elements (Fig. 3, Additional file 7: File S3), many of which are simple/satellite (10.9% of the genome) or low complexity repeats (5.6% of the genome). We also found all other classes of repetitive elements as in the *B. plicatilis* genomes in this genome, including SINE elements (0.26 Mbp, or 0.08% of the genome), which were not previously reported.

### Gene annotations

We used the protein sequences of the predicted gene models from the published *B. calyciflorus* genome [32] to annotate 11,000–12,500 genes in each of our five genome assemblies (Table 2). The assemblies had fewer annotated genes than the *B. calyciflorus* reference. The difference in gene number could be accounted for due to our assemblies all having far fewer single-intron genes. Our assemblies also have smaller mean lengths of exons, introns, and intergenic regions. A smaller mean intergenic distance could be an artefact of a less-contiguous assembly, so intergenic distance for *B. calyciflorus* was recalculated as if each contig was broken in 10 pieces, however, this did not reduce the intergenic distance (not shown). In contrast, our assemblies had a higher proportion of pseudogenes than *B. calyciflorus*, and the number of pseudogenes increased with genome size (R^2^ = 0.93). In the species with smaller genomes (*B. rotundiformis*, *B*. sp. ‘Tiscar’, and *B. plicatilis*), mean intron size increased with genome size (R^2^ = 0.95), resulting in an increase in total intronic DNA. However, the total contribution of pseudogenes and intronic DNA is relatively small compared to overall differences in genome size.

**Table 2:** Gene number after annotation and quality filtering with fathom, the number of single exon genes, number of potential pseudogenes, sum total gene, exon and intron sizes, mean exon and intron size, mean intergenic size, intergenic50 (similar to N50, but calculated with intergenic size instead of contig size), and the GC content of the genes

Species name	*B. rotundiformis*	*B.* sp. ‘Tiscar’	*B. plicatilis*	*B.calycifloris*	*B. asplanchnoidis*
Genome size (Mbp)	110	164	246	293	422
Gene number	11,050	12,085	12,484	15,628	12,547
Number of single exon genes	257	323	460	4321	12
Number of potential pseudogenes	1441	2072	2313	481	2953
Sum total gene size (Mbp)	25.89	29.82	32.03	43.77	30.11
Sum total exon size (Mbp)	16.35	16.52	16.21	24.98	15.35
Sum total intron size (Mbp)	9.53	13.29	15.82	18.79	14.76
Mean exon size (bp)	181	173	175	394	180
Mean intron size (bp)	118	157	194	392	199
Mean intergenic size (bp)	2059	1832	2059	5051	1872
Median intergenic size (bp)	1052	944	1052	2746	925
Intergenic50	4206	4478	5017	10,103	4455
GC % in genes	29.2	27.5	28.4	26.4	27.7

Most of the annotated genes, when clustered by OrthoVenn, were shared between all, or most of the assemblies. Only 446 of 12,372 gene clusters were found in any single assembly and not shared by any others (Additional file 4: Figure S4). Most of these gene clusters (366) were in the *B. calyciflorus* genome assembly. The *B. calyciflorus* genome assembly also had about 1000 more gene clusters than the *B. plicatilis* genomes annotated here.

## Discussion

### Genome sequencing, assembly and assessment

Here, we present assemblies of five genomes from four species of the *Brachionus plicatilis* species complex, which we have compared to a recently published genome from the same genus [32]. Our sequencing libraries had relatively low contamination levels (0.4–7.9%). Nevertheless, assembly statistics showed improvement, with most N50 s doubling, after removal of these contaminants (even when only 0.4% of the reads were removed; Table 1), supporting the necessity of this step in whole-genome sequencing [33]. After removing contaminants, estimated 1n genome coverage ranged from 17x-33x. Our assemblies were relatively complete, in terms of genic regions (as shown by BUSCO gene annotations and whole genome gene annotations). Out of the 978 metazoan BUSCO genes, 5.5% were missing from all of our assemblies, suggesting that they are likely absent from the genomes of these species. Due to the sequencing strategy of short, paired-end reads, the assemblies presented here were more fragmented than the *B. calyciflorus* genome assembly [32], and likely incomplete in terms of repetitive element content. The more fragmented assemblies and higher proportion of unmapped reads in the larger genomes indicates that the unassembled regions likely consist of mostly repetitive elements [34, 35].

### Polyploidy

Polyploidisation is a powerful evolutionary force, driving drastic changes in genome size [1, 36, 37], influencing speciation [38, 39], and generating evolutionary novelties [40]. Other rotifer species have been found to be polyploid [41–43], so here we considered evidence for the role of polyploidy in the large interspecific genome size variation in *B. plicatilis*. Perhaps surprisingly, we found no strong evidence that ploidy variation drives genome size change in the species complex (Fig. 2, Additional file 1: Figure S1 and Additional file 2: Figure S2). In *B. calyciflorus*, most kmer pairs indicated diploidy, but some triploid and tetraploid regions were also detected. This might indicate a hybridisation event in the past that has been followed by rediploidization. Recent, or even ongoing, hybridisation has been previously proposed in *B. calyciflorus* [44], so it is possible that the sequenced *B. calyciflorus* clone represents one of these recently-hybridised individuals.

### Repetitive element expansion and activity

**Fig. 5:**
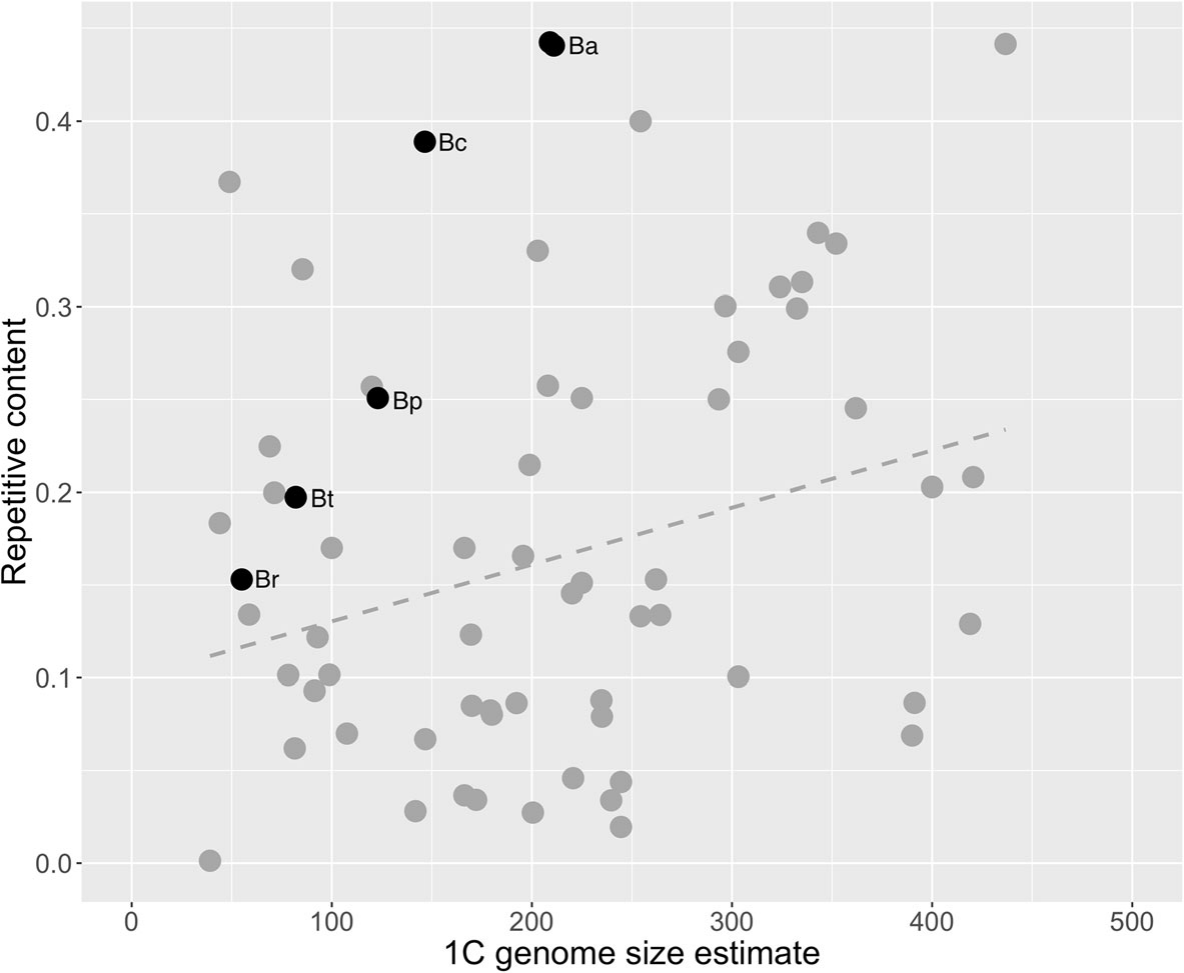
The repetitive content of the five *Brachionus* genomes presented here (black points) compared to animal genomes (grey points, *n* = 59, *p* = 0.0202, R^2^ = 0.075) with less than 500 Mbp 1C genome size and their repeat content or transposable element content estimates. All data from [3]. *Brachionus* 1C genome estimates were calculated assuming diploidy (i.e. genome size/2). Labels next to the *Brachionus* points indicate species names (Br = *B. rotundiformis*, Bt = *B.* sp. ‘Tiscar’*,* Bp = *B. plicatilis*, Bc = *B. calyciflorus*, Ba = *B. asplanchnoidis*)

Across the *Brachionus* genus, and the *B. plicatilis* species complex, repetitive elements clearly increased with genome size (Fig. 3), confirming similar trends observed in other animal taxa (e.g., [18]). This was evident across all repeat element annotation methods used. Repetitive element estimates from de novo annotation of read libraries (both proportional and in Mbp) correlated significantly with genome size, and could explain up to 71% of the genome size change across species in this species complex. The relative contribution of the least diverged LTR, LINE, and DNA elements vary even within a single species (Fig. 4), suggesting this process may be ongoing. When compared to other animal genomes of similar size (Fig. 5), it is clear that *Brachionus* genomes contain remarkably high proportions of repetitive DNA. This is especially obvious when considering the 150 and 210 Mbp genomes of *B. calyciflorus* and *B. asplanchnoidis* genomes, which contain 39 and 44% repetitive elements.

Asexuality is potentially linked to lower repetitive element burden [45], but monogonont rotifers are cyclical parthenogens. Thus, one might argue that it is even more surprising that the genomes of our studied species contain such high proportions of repetitive DNA. However, given that *Brachionus* and other monogonont rotifers regularly engage in sex, but not every generation, we would not expect them to carry genomic signatures of long-term obligate asexuality.

The estimated contribution of repetitive elements to each genome assembly varied by annotation method, and especially between read-based and assembly-based strategies. When annotating repeats with assembly-based methods (especially when relying on existing databases; Additional file 7: File S3), repetitive content estimates were very low, and likely represented underestimates due to not accounting for novel repetitive elements, assembly coverage or unassembled regions [34, 46]. The method used for de novo repeat annotation of read libraries considers these factors, though may still underestimate repetitive content [31]. These differences in repeat annotations were very clear when comparing our repetitive content estimates of *B. calyciflorus* (38.9%) to the published estimate (21%), which was based on assembly annotation [32]. It is thus clear that relying on database and genome assembly approaches for repeat annotation in non-model organisms is insufficient [31, 46]. Despite these annotation improvements from short read sequencing data, confirmation of repeat structure through long read sequencing followed by manual curation, especially of the unclassified repeats, would provide the most confident repetitive element annotations for more detailed analyses [47].

### Gene annotation

Annotated gene content across all our genome assemblies was similar, but lower than the number of genes previously annotated in the *B. calyciflorus* genome (Table 2)*.* The previous annotation of the *B. calyciflorus* genome included an order of magnitude more single-exon genes than annotations of our assemblies, which accounts for the differences in total gene number. Retrotransposition could be creating these single exon genes [48], although it would be surprising if this were confined to *B. calyciflorus*. Further validation of these genes with transcriptome evidence across all species, and identical annotation methods, would confirm if these differences are real. Gene orthology analyses (Additional file 4: Figure S4) between the species suggests that the rest of the gene annotations of our assemblies were representative of the genes and gene families identified in *B. calyciflorus*. The *B. calyciflorus* genome assembly has larger intergenic distances, even when we simulated assembly fragmentation. However, this simulated assembly fragmentation was not random and did not account for where short-read assemblies would normally be broken (in highly repetitive regions). The number of pseudogenes increased with genome size, with the largest genomes (*B. asplanchnoidis*) having twice the number of pseudogenes as the smallest genome (Italy2). While this increase is not a significant contribution to the differences in genome size, it is consistent with the increase in retrotransposable element load [49, 50], and provides additional evidence that repeat element proliferation has played a role in genome size variation in the *B. plicatilis* species complex. RNASeq mapping of genes and more contiguous genome assemblies of the *B. plicatilis* species would improve annotation and provide the basis for exploring gene evolution across the *Brachionus* genus, especially investigations into the links between polyploidy and speciation, gene loss or gene family expansion [42, 43].

## Conclusions

We have analysed the genomes of four of the species in the *B. plicatilis* species complex, which span much of the range of genome sizes observed in this complex. Overall, we identified a high proportion of repetitive elements in these genomes (14–44%), much higher than most animal genomes of similar size. There is some evidence for recent accumulation of LINE elements, DNA transposons and LTRs, which may be contributing actively to genome expansion. Additionally, we identified almost twice as many repetitive elements as previously reported in the *B. calyciflorus* genome, showing the utility of read-based de novo repeat annotation. Transposable element activity clearly plays a role in genome evolution and expansion in the *B. plicatilis* complex, but polyploidy does not appear to contribute to genome size differences across this species complex. This species complex represents a valuable model to study the dramatic impacts transposable elements can have on genomes.

## Methods

### Animal culture genome sequencing, assembly and assessment

In this study, we used clones from *B. rotundiformis* (Italy2), *B.* sp.*’*Tiscar*’* (TiscarSM28), *B. plicatilis* s.s. (Tokyo1) and two *B. asplanchnoidis* clones (OHJ82 and OHJ22) previously described [22]. Rotifer clonal populations were maintained and cultured following previous protocols [23]. Rotifers were cultured in F/2 medium [51] at 16 ppt salinity and fed *Tetraselmis suecica* algae at ad libitum concentration (500–1000 cells μl^− 1^).

DNA extraction methods followed those in a previous study [23]. In order to ensure enough rotifer biomass for DNA extraction, the clonal cultures were grown to a density of 10–100 individuals per ml. To reduce contamination by DNA from the food algae, the cultures were starved for 16 h, ensuring that rotifers completely emptied their guts. The DNeasy Blood & Tissue kit (Qiagen) was used to isolate genomic DNA according to the manufacturer’s instructions, except that DNA was eluted with 50 μl of TE0.1 buffer (20 mM Tris–HCl, 0.1 mM EDTA, pH 8.0). DNA quality and concentration were checked by running a 1% agarose gel and measured with a NanoDrop spectrophotmeter (Thermo Scientific).

Italy2, TiscarSM28, Tokyo1, and OHJ22 genomic libraries were prepared from 450 ng DNA with KAPA HyperPlus Library Preparation Kit (Kapa Biosystems, Wilmington, MA, USA). The OHJ82 library was prepared from 1 μg DNA using the KAPA Hyper Prep Kit after shearing by Covaris S220 and AFA microtubes (Covaris, Woburn, MA, USA) All libraries were ligated to Illumina TruSeq Indexed Adapters (IDT, Coralville, IA, USA), and subjected to a single cycle of PCR to prepare fully double-stranded fragments, prior to size selection and quality assessment with Bioanalyzer High Sensitivity DNA Kit (Agilent, Santa Clara, CA). Libraries were quantified by Quant-iT™ PicoGreen® dsDNA Assay Kit (Thermo Scientific, Waltham, MA, USA), and equimolar amounts were pooled and concentrated with MinElute PCR Purification Kit (Qiagen, Germantown, MD, USA) prior to tight size selection at 450 bp with Pippin Prep 1.5% cassette (Sage Science, Beverly, MA, USA). The final, pooled, size-selected samples were cleaned with MinElute, assessed again by Bioanalyzer High Sensitivity DNA Kit, and quantified by qPCR using KAPA Library Quant Kit for Illumina.

Paired-end sequencing was done on Illumina HiSeq 1000 (2x125bp) and /or on Illumina NextSeq (2x150bp) platform at the MBL’s W. M. Keck Ecological and Evolutionary Genetics Facility until coverage was estimated to be ≥15x.

Reads were quality filtered [52] and assembled by CLC Workbench V7 (Qiagen). CLC Assemblies were done with the following settings: minimum contig length 500 bp, mismatch cost 2, insertion cost 3, deletion cost 3, length fraction 0.8, similarity fraction 0.93. Once genome assemblies were generated, the raw filtered reads from each genome were mapped back to their respective genomes. All mapping was performed with bowtie2 [53] under default parameters.

Contaminant contigs (mostly of bacterial origin) were identified using Blobtools v1.0 [33] using a GC-dependent coverage cut-off. Read pairs that both mapped back to a contaminant contig using bowtie2 were classed as contaminants. All other reads were reassembled using SPAdes v3.12.0 [54] with default settings. This process was repeated twice, and the final uncontaminated assemblies were screened once more, and contaminant reads and contigs were removed again, but not reassembled. All further analyses were performed using these uncontaminated assemblies and read libraries. BUSCO v2 [55] was used to annotate each uncontaminated genome assembly using the metazoan_obd9 database (978 genes). These regions were then compared between assemblies, and shared regions were used for further analyses. The *Brachionus calyciflorus* PE500 library [32] was downloaded from NCBI (SRA SRR6027265), and the same assembly and cleaning procedure was followed except that contigs with best blast hits only to bacteria in the blobtools pipeline were removed regardless of GC content or coverage. After contaminant removal, the *B. calyciflorus* library contained 51,092,536 read pairs (25.6 Gbp, 73.3% of the raw reads). These cleaned read libraries were then used for further analyses.

### Repeat content estimates

RepeatMasker v 4.0.6 [29] was used on the genomes sequenced here with the species option specifying “metazoa” and the NCBI search engine. Additionally, the program dnaPipeTE v1.3 [31] was used to assemble and assess the repetitive content of the *B. plicatilis* and *B. calyciflorus* genome (for this, only the decontaminated PE500 read library detailed above was used). Briefly, dnaPipeTE subsamples the short-read sequencing libraries at low genome coverage and assembles each sample of reads with Trinity (so that repeat element copies are grouped together like transcript isoforms), the assemblies from each sample are compared, consolidated, and annotated with RepeatMasker, RepBase and BLAST, and a sample of reads is compared to this consolidated set of repeat element contigs via BLASTn to determine which proportions of the genome are repetitive and which are low-copy. This results in an estimate of genome contributions (as proportions) from different types of genomic elements, including low-copy DNA, transposon classes like Long-Terminal Repeats (LTRs), Long and Short Interspersed Elements (LINEs and SINEs), DNA transposons, Miniature Inverted-repeat Transposable Elements (MITEs) and other repetitive elements such as Ribosomal RNA, low complexity sequences (such as AT rich regions) and simple/tandem repeats (satellite DNA). This pipeline also gives an indication of the relative age of repetitive elements through the percent identity results from one of the BLAST searches. We used dnaPipeTE with 10 subsamples at 0.05x coverage (of genome size), ensuring that most repetitive elements were assembled in all cases. The dnaPipeTE contigs were then also used as custom libraries for RepeatMasker. RepeatModeler v1.0.11 [30] with default options was used for de novo annotation of repetitive elements in each genome assembly using a databse built from that assembly. These sequences were then also used as custom RepeatMasker libraries.

The dnaPipeTE output and dnaPipeTE + RepeatMasker output were compared, both for size (in Mbp) of repetitive regions in the genome assemblies, and divergence estimates for each class of repetitive element. Then, to determine if the number of repetitive elements at any divergence-level of particular in each class correlated with genome size, the count of each bin (bin sizes used- 2, 5, 10%) was determined for each genome and a linear regression was performed, *p* values were Bonferroni corrected for multiple testing.

### Ploidy analysis

Jellyfish v2.1.4 [56] was used to extract kmers and kmer coverage histograms from decontaminated read libraries (k21, coverage limits for kmer extraction were set above the error rate for each read library, with a maximum coverage of 200x to minimise noise from repetitive regions). GenomeScope [57] was used to estimate error rates, heterozygosity, and to estimate 1n kmer coverage of each read library, and smudgeplot v0.1.3 (available at https://github.com/tbenavi1/smudgeplot) was used to identify kmer pairs with exactly one difference between them, and then the coverage of each kmer pair and the relative coverage of the minor kmer compared to total kmer pair coverage were plotted in 2D distribution plots. Smudgeplot was allowed to estimate the 1n coverage freely, unless the 1n coverage estimate differed greatly from both the genome sequencing coverage estimates (Table 1) and the GenomeScope 1n coverage estimate. The coverage of the whole assemblies and the previously identified shared single copy orthologues was compared across all genomes. Average read depth over the whole genome (in 500 bp windows) and regions of interest was calculated using samtools v1.9 [58].

The shared BUSCO genes were also used to assess allele frequencies. Freebayes v1.1.0–54-g49413aa [59] was used to identify SNPs and extract the number of reads which mapped to each variant (mapping quality 30, read quality 20, minimum coverage 5). Allele frequencies were calculated from the proportions of reads which map to each variant. The package nQuire [60] was used to assess allele frequency distributions in the whole assemblies up to 200x coverage. It was also used to assess allele frequency distribution in all genes in the *B. calyciflorus* genome assembly. For all nQuire analyses, a minimum mapping quality of 30, and a maximum coverage of 200 were used.

### Gene annotation

One masked assembly per species from the dnaPipeTE masking (above) was used for gene annotation using MAKER v 2.31.10 [61]. For *B. asplanchnoidis*, genome assembly OHJ22 was used. The protein sequences from the recently published *B. calicyflorus* genome were used to generate a gene model for each assembly. This gene model was used to train SNAP within MAKER, and the output of this was then used again to train SNAP for a more refined gene model. These gene models were then used for further analysis using fathom, gffread v0.10.1, and custom scripts in R v3.5.1. Proteins from these annotations were compared using OrthoVenn [62].

## Additional files

Additional file 1: **Figure S1.** Allele frequency distribution of the 740 shared BUSCO genes in each assembly. Grey bars indicate allele frequency relative counts (bin width = 0.01) of these frequencies. (PNG 32 kb)

Additional file 2: **Figure S2.** Distributions of total coverage of heterozygous k21 kmer pairs and normalised minor kmer coverage plotted together for read libraries for each genome. (PNG 338 kb)

Additional file 3: **Figure S3.** Relative distributions of Kimura distance estimates of three repetitive element classes from repetitive element annotation of read libraries (red) and assemblies (blue). (PNG 273 kb)

Additional file 4: **Figure S4.** A diagram showing the overlapping gene clusters in each genome assembly (from OrthoVenn), the number of gene clusters in each assembly, and the number of clusters shared between the different numbers of assemblies. (PNG 253 kb)

Additional file 5: **File S1.** A table of complete, duplicated, fragmented, and missing BUSCO genes for each of the genomes sequenced here and the average coverage of each of the shared BUSCO genes for each assembly. (XLSX 284 kb)

Additional file 6: **File S2.** A summary of log-likelihood scores from the nQuire programme, showing the percent of heterozygous sites retained after the denoise step, the score for the free, diploid, triploid, and tetraploid models, and the differences between the diploid, triploid, and tetraploid models and the free model. The “Allreads_nQuire” tab summarises the results for when all decontaminated reads were mapped back to the genome assemblies, “Repfree_selfmaps_nQuire” summarises the results for when repetitive reads were removed from the read libraries (all reads were mapped to the repeats assembled by dnaPipeTE, and read pairs where neither read mapped to the repeats were extracted and considered “repeat free”) and mapped back to the assemblies, and “Repfree_reads_BUSCO_nQuire” summarises the same repeat free mappings, but restricted only to the shared 740 BUSCO genes. (XLSX 12 kb)

Additional file 7: **File S3.** A summary of the repetitive content estimates from each method. “dnaPipeTE_proportions” are the proportional estimates derived from the dnaPipeTE pipeline applied to read libraries, “dnaPipeTE_Mbp_fromprop” reflects these proportional estimates in Mbp (i.e. for each genome, the proportion of each element * the genome size), “dnaPipeTE_Mbp_RM” is the Mbp of each assembly which was masked by RepeatMasker using the dnaPipeTE repetitive element assemblies as repeat libraries, “RepeatModeler_Mbp_RM” when the RepeatModeler libraries were used for masking the assemblies with RepeatMasker, and “RM_metazoa_Mbp” the Mbp of each assembly which were masked with the “metazoa” library of RepeatMasker. (XLSX 16 kb)

## Data Availability

Raw read files, final assemblies and cleaned reads mapped to final assemblies can be found, with all accession numbers, on NCBI under BioProject PRJNA541909.

## Abbreviations

bp: Base pairs
Gbp: Gigabase pairs
LINE: Long Interspersed Nuclear Elements
LTR: Long Terminal Repeats
Mbp: Megabase pairs
SINE: Short Interspersed Nuclear Element
SNP: Single nucleotide polymorphism
