# Supplementary figures and images for: Small, but surprisingly repetitive genomes: transposon expansion and not polyploidy has driven a doubling in genome size in a metazoan species complex
Source: BMC Genomics. 2019 Jun 7;20:466. doi: 10.1186/s12864-019-5859-y (PMC6555955; doi:10.1186/s12864-019-5859-y)

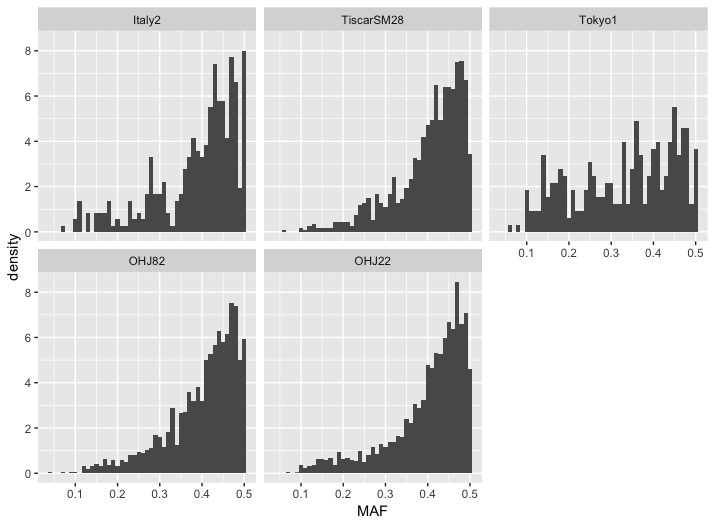

Supplement: Supplementary file 1 — Figure S1. Allele frequency distribution of the 740 shared BUSCO genes in each assembly. Grey bars indicate allele frequency relative counts (bin width = 0.01) of these frequencies. (PNG 32 kb) [file 12864_2019_5859_MOESM1_ESM.png]

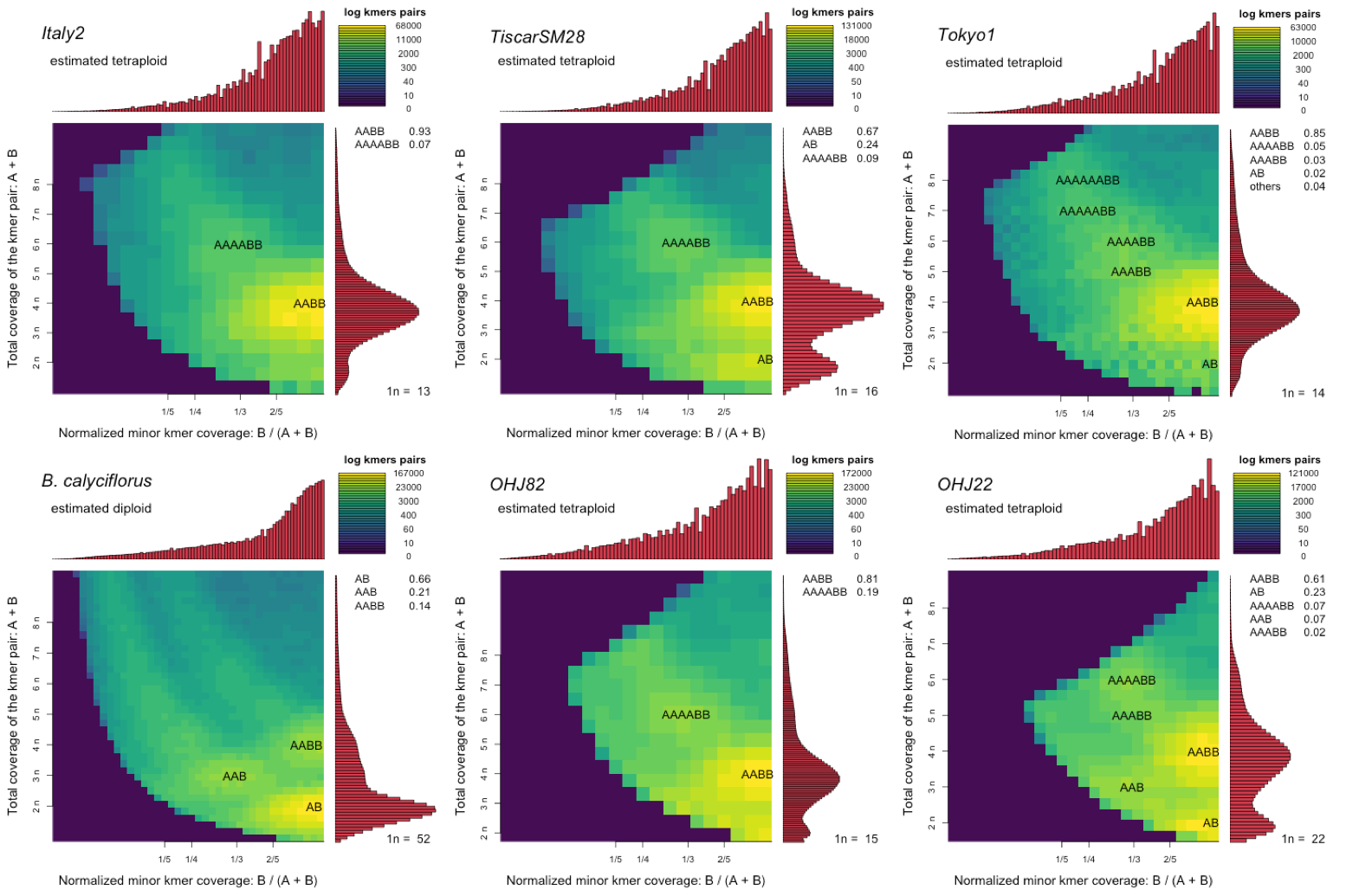

Supplement: Supplementary file 2 — Figure S2. Distributions of total coverage of heterozygous k21 kmer pairs and normalised minor kmer coverage plotted together for read libraries for each genome. (PNG 338 kb) [file 12864_2019_5859_MOESM2_ESM.png]

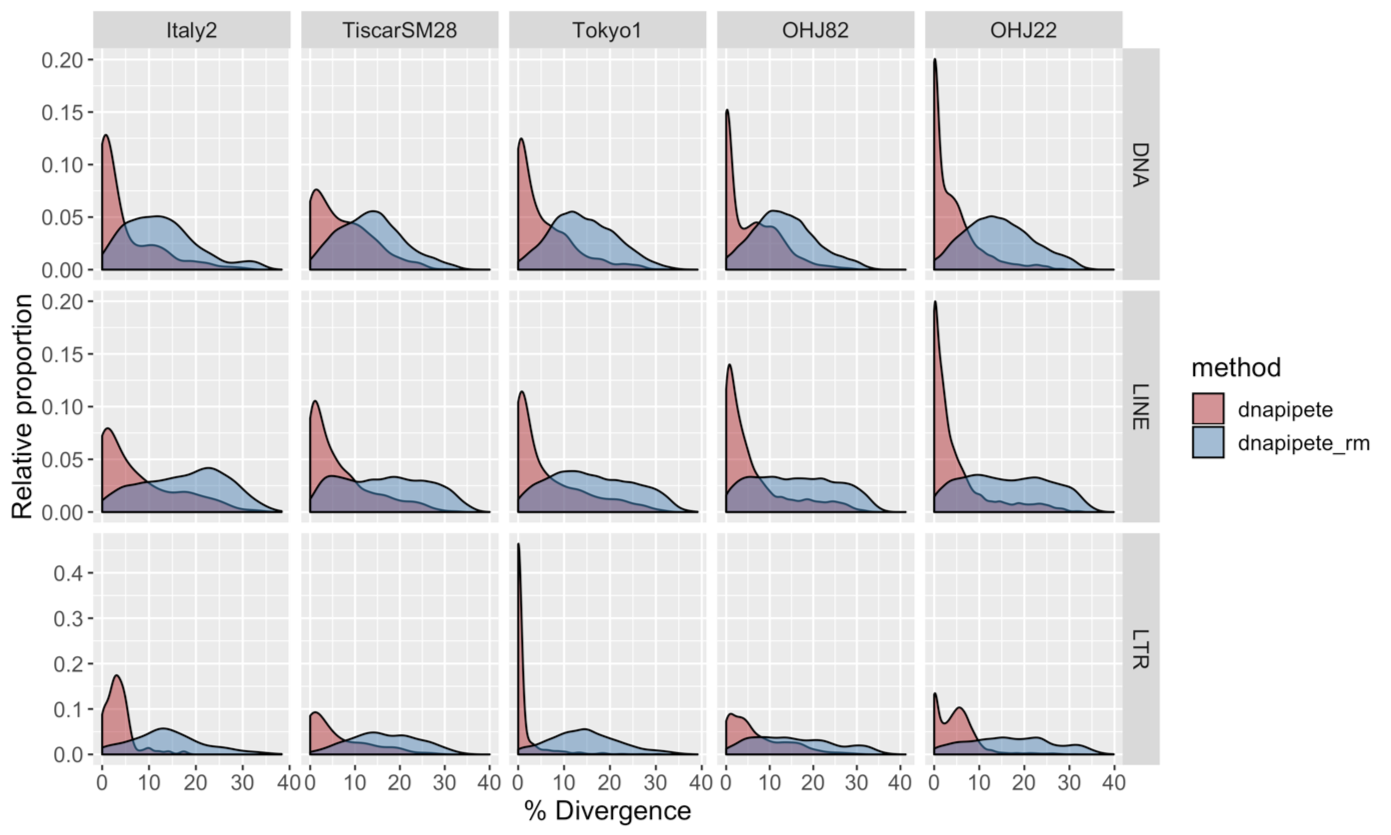

Supplement: Supplementary file 3 — Figure S3. Relative distributions of Kimura distance estimates of three repetitive element classes from repetitive element annotation of read libraries (red) and assemblies (blue). (PNG 273 kb) [file 12864_2019_5859_MOESM3_ESM.png]

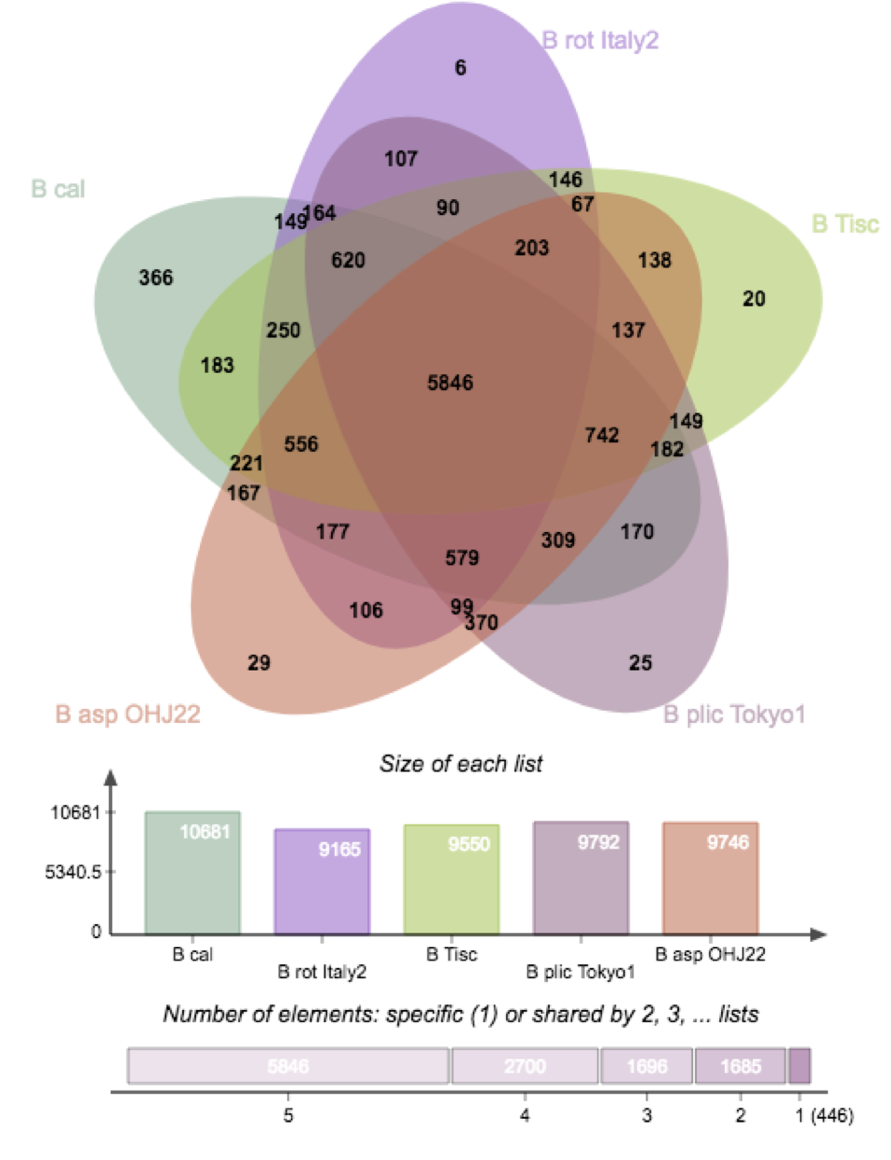

Supplement: Supplementary file 4 — Figure S4. A diagram showing the overlapping gene clusters in each genome assembly (from OrthoVenn), the number of gene clusters in each assembly, and the number of clusters shared between the different numbers of assemblies. (PNG 253 kb) [file 12864_2019_5859_MOESM4_ESM.png]
